# Comparison of classification methods that combine clinical data and high-dimensional mass spectrometry data

**DOI:** 10.1186/s12859-014-0385-z

**Published:** 2014-11-29

**Authors:** Caroline Truntzer, Elise Mostacci, Aline Jeannin, Jean-Michel Petit, Patrick Ducoroy, Hervé Cardot

**Affiliations:** Proteomic Platform CLIPP, Centre Hospitalier Universitaire, Dijon, 21000 France; Service Endocrinologie, Centre Hospitalier Universitaire, Dijon, 21000 France; Institut de Mathématiques de Bourgogne, UMR CNRS 5584, Dijon, 21000 France; University of Burgundy, Dijon, 21000 France

**Keywords:** High-dimension, Predictive value, Biomarkers, Classification methods, Clinical data

## Abstract

**Background:**

The identification of new diagnostic or prognostic biomarkers is one of the main aims of clinical cancer research. Technologies like mass spectrometry are commonly being used in proteomic research. Mass spectrometry signals show the proteomic profiles of the individuals under study at a given time. These profiles correspond to the recording of a large number of proteins, much larger than the number of individuals. These variables come in addition to or to complete classical clinical variables. The objective of this study is to evaluate and compare the predictive ability of new and existing models combining mass spectrometry data and classical clinical variables. This study was conducted in the context of binary prediction.

**Results:**

To achieve this goal, simulated data as well as a real dataset dedicated to the selection of proteomic markers of steatosis were used to evaluate the methods. The proposed methods meet the challenge of high-dimensional data and the selection of predictive markers by using penalization methods (Ridge, Lasso) and dimension reduction techniques (PLS), as well as a combination of both strategies through sparse PLS in the context of a binary class prediction. The methods were compared in terms of mean classification rate and their ability to select the true predictive values. These comparisons were done on clinical-only models, mass-spectrometry-only models and combined models.

**Conclusions:**

It was shown that models which combine both types of data can be more efficient than models that use only clinical or mass spectrometry data when the sample size of the dataset is large enough.

## Background

The search for relevant proteins or biomarkers is a main issue in clinical research. Proteins that make up the proteome are representative of the cellular state and at a bigger scale of the patient’s health. The discovery of new biomarkers would lead to more accurate diagnosis or prognosis. Technologies like mass spectrometry (MS) are commonly being used in clinical proteomic research. This technology allows the separation and large-scale detection of proteins present in a complex biological mixture. For each biological sample, the MS signal shows relative protein abundance according to their molecular weight-over-charge. The acquisition measurements reflect the proteomic profiles of the individuals under study at a given time.

MS signals consist in the recording of *p* protein intensities for each of the *n* individuals under study. In proteomic studies, the number *p* of recorded variables is high compared with the number *n* of individuals, *n*≪*p*.By analyzing these proteomic profiles, researchers are looking for biomarkers that allow, for example, the classification of samples to distinguish between two groups such as healthy and sick individuals or relapse-free and relapsed patients. In the context of clinical research, classical clinical variables like age, sex, or duration of the disease, for example, are already used routinely for such classifications. The idea in this paper was to evaluate the predictive contribution given by MS data when combined with classical clinical variables. To this end, models that combine both types of variables and that improve predictions given the specificities of each ([1,2]) have to be constructed. Classical clinical variables have already been identified and validated through many previous studies. High-dimensional MS data, however, are still being identified, and high dimension analysis is still a challenge in statistics. In this paper, we propose to compare different models using 1) clinical data, 2) MS data and 3) a combination of both clinical and MS data. Clinical and MS variables are combined in different ways to determine the best way to introduce them into a single model in terms of misclassification rate and ability to detect truepositives.

A classical tool for binary classification with clinical data is logistic regression. Because of the multicollinearity problem, the high dimension of MS data makes it impossible to use logistic regression directly. In regression, the system of equations becomes singular and the solution, when it exists, is not unique. To overcome this problem, it is necessary to add constraints in the model.

There exist mainly two kinds of approaches that have been developed to handle this issue: penalization methods or dimension reduction methods [3-5].

Penalization methods like ridge or lasso regression consist in maximizing the log-likelihood under constraint on the set of parameter estimates. This results in shrinkage of the estimated parameters. Dimension reduction methods handle the multicollinearity by projecting the data into a lower dimension space defined by new variables, called components. These two approaches can also be combined into a single approach by penalizing the coefficients of the components. Each of these three approaches can be performed through different methods. The goal here is not to make a complet review of all existing methods but to comment some specific ones. The selected methods were chosen because they are popular in bioinformatics and high dimensional statistics, and representative of these three kinds of approaches.

Among the penalization methods, ridge regression [6] shrinks the parameter estimates through an $\mathcal {L}_{2}$ penalty. Lasso regression [7] shrinks the parameter estimates through an $\mathcal {L}_{1}$ penalty, thus imposing a penalty on their absolute values. This constraint sets certain parameters exactly to zero, which leads to the selection of the most predictive variables. The elastic net method proposed by Zou and Hastie [8] combines the properties of both $\mathcal {L}_{2}$ and $\mathcal {L}_{1}$ penalties. Furthermore, boosting is another method that can be related to the Lasso as it also uses a penalty strategy that leads to variable selection. This general framework is defined by a loss function and a base procedure [9-12]. Considering the component-wise-linear-squares base procedure and the negative log-likelihood loss function, boosting can be used to fit high-dimensional models with a binary response.

Another interesting alternative to handle the multicollinearity issue is to reduce the data dimension while keeping the relevant features. In this case, dimension reduction is achieved by projecting the data into a lower dimension space defined by new variables, called components. These components are constructed as linear combinations of the original variables.

As for dimension reduction, Partial Least Squares (PLS) [13-15] builds components so as to maximize the covariance between the response and the components. PLS was initially designed for continuous responses. To adapt PLS to classification problems, Fort and Lambert-Lacroix [16] proposed the Ridge PLS (RPLS) method, which uses penalized logistic regression and PLS for binary responses. This algorithm combines a regularization step and a dimension reduction step. However, it does not provide variable selection, making it difficult to interpret results in terms of biomarker discovery. To produce more interpretable results, Chung and Keles proposed a Sparse Generalized PLS (SGPLS) method. SGPLS provides PLS-based classification with variable selection, by incorporating sparse partial least squares (SPLS) [17] into a generalized linear model (GLM) framework. Instead of being linear combinations of all of the variables, SGPLS components are linear combinations of a subset of the variables selected through an elastic net constraint [8].

In order to assess a model that combines both clinical and high-dimensional data, Boulesteix and Hothorn [18] suggested a two-step procedure using a logistic model to estimate the parameters of the clinical variables in step 1 and a boosting algorithm to estimate the parameters of the high-dimensional data in step 2. We propose to extend this idea to the RPLS and SGPLS algorithms. More precisely, we propose in this paper two procedures called RPLSOffClin (RPLS with the clinical predictor as an offset) and SGPLSOffClin (SGPLS with the clinical predictor as an offset) defined as follows: 1) build a clinical predictor using logistic regression, 2) run a modified RPLS or SGPLS algorithm to take into account the clinical predictor as an offset.

The paper is organized as follows. In the first section we present a brief review of GLM in the case of binary classification, boosting and PLS dimension reduction methods. We then present the RPLS and SGPLS algorithms and the extensions we propose to include clinical information. In the second section these different methods are compared in terms of their predictive accuracy on both simulated and real datasets.

## Methods

### Notations

Each of the *n* individuals under study is described by his proteomics and clinical information. The *n*×*p* matrix **X**=(*x*_*ij*_), *i*=1,…,*n*, *j*=1,…,*p* (*n*≪*p*), contains the intensities of the *p* proteins. The *n*×*q* matrix **Z**=(*z*_*ik*_), *i*=1,…,*n*, *k*=1,…,*q* (*q*<*n*) contains the clinical variables. The notation *x*_*i*._ (resp. *z*_*i*._) corresponds to the recording of all of the MS (resp. clinical) variables for each *i*^*th*^ individual. The recording of the *j*^*th*^ MS (resp. clinical) variable for all individuals is denoted *x*_.*j*_ (resp. *z*_.*j*_). The response variable *y* is coded as *y*∈{0,1} with realizations *y*_1_,…,*y*_*n*_.

### Generalized linear models (GLM)

The GLM model can be written as [19]:

(1) $${} g(E(y_{i}))=\eta_{i}(\gamma)=\gamma_{0}+\gamma_{1}z_{i1}+\ldots+ \gamma_{q}z_{iq},\; i=1,\ldots,n  $$

*z*_*ij*_ corresponds to the *j*^*th*^ clinical variable for the *i*^*th*^ individual under study; *y*_*i*_ corresponds to the response of interest. In case of a binary *y* response (healthy or diseased for example, coded as *y*∈{0,1}), the logit function is chosen as the link function.

$$logit(\pi_{i})=\text{log}\left(\frac{\pi_{i}}{1-\pi_{i}}\right),\; i=1,\ldots,n $$

with *π*=*P*(*y*=1|**Z**).

The unknown parameters (*γ*_0_,…,*γ*_*q*_)=*γ*∈**R**^*q*+1^ are generally estimated by maximizing the log-likelihood function given by

(2) $$\begin{array}{@{}rcl@{}} l(\gamma)=\sum_{i=1}^{n}\left(y_{i}z_{i.}\gamma-\text{log}\left(1+e^{z_{i.}\gamma}\right)\right) \end{array} $$

The maximum likelihood (ML) estimators $\hat {\gamma }$ are solutions of the log-likelihood equations

(3) $$\begin{array}{@{}rcl@{}} \frac{\partial l(\gamma\!)}{\partial \gamma_{k}}=\sum_{i=1}^{n}z'_{ik}\!\left(\hspace*{1pt}y_{i}-\pi_{i}\right)=0,\; k=1,\ldots,q \end{array} $$

Because of the non-linearity of these equations, explicit solutions cannot be obtained. To solve this system, the Newton-Raphson algorithm can be used. This Iteratively Reweighted Least Squares (IRLS) algorithm [20] determines the solution by successive approximations of *γ* until convergence.

At step *h*, the ML estimators verify the following updating step

(4) $$\begin{array}{@{}rcl@{}} \hat{\gamma}^{h+1}=\left(\mathbf{Z}'\mathbf{W}^{h}\mathbf{Z}\right)^{-1}\mathbf{Z}'\mathbf{W}^{h}\tilde{y}^{h} \end{array} $$

where $\tilde {y}^{h}=\mathbf {Z}\gamma ^{h}+\frac {y-\pi ^{h}}{\mathbf {W}^{h}}$ is the pseudo-response and the diagonal entries of the weight matrix **W**^*h*^ are ${w_{i}^{h}}={\pi _{i}^{h}}\left (1-{\pi _{i}^{h}}\right)$. Each step of the algorithm is a least square regression of $\tilde {y}^{h}$ on **Z** weighted by **W**^*h*^. At convergence, the pseudo-variable is denoted by $\tilde {y}^{*}$ and the weight matrix by **W**^∗^.

### Boosting

Boosting is an iterative stepwise gradient descent algorithm [10,21,22]. The principle of boosting is to improve the performance of a regression method by iteratively fitting a base procedure to the residuals. At each step *h* the base procedure aims at constructing a function $\hat {g}^{h}$ which is used to build the final predictor $\hat {f}$ as a linear combination of the $\hat {g}^{h}$ function estimates.

In the case of high-dimensional data with a binary response, the following loss-function is considered [12]

(5) $$\begin{array}{@{}rcl@{}} \rho(y,f)=\log\left(1+e^{-2\hat{y}f}\right) \end{array} $$

where $\hat {y}=2y-1\in \{-1;+1\}$ and *f*=*log*(*π*/(1−*π*))/2.

The base procedure is chosen as a component-wise linear least squares procedure. The main steps are described below.

1. Initialization: *h*=0, $\hat {f}^{0}=\text {log}\left (\frac {\pi }{1-\pi }\right)/2$, where $\pi =\mathbb {P}(\mathbf {y}=1|\mathbf {X})$, $\hat {\beta }^{0}=0$.
2. Calculate the negative gradient of the loss function: *h*=*h*+1, $u_{i}=-\frac {\partial }{\partial f}\rho (y_{i},f)\vert _{f^{h-1}(x_{i.})}$, *i*=1,…,*n*.
3. Component-wise Linear Least Squares base-procedure:
  Define $\hat {\beta }^{hj}$, the jth component of $\hat {\beta ^{h}}$ (size *p*), as $\hat {\beta }^{j}=\sum _{i=1}^{n} x_{\textit {ij}}u_{i}/\sum _{i=1}^{n}(x_{\textit {ij}})^{2}$
  Select among the *p* variables the one that minimizes the error $\sum _{i=1}^{n}\left (u_{i}-\hat {\beta }^{j}x_{\textit {ij}}\right)^{2}$. Let $x_{.s_{h}}$ be the selected variable at step *h*.
  Build the estimate function $\hat {g}_{i}^{h}=\hat {\beta }^{s_{h}}x_{is_{h}}$, *i*=1,…,*n*.
4. Update $\hat {f}^{h}=\hat {f}^{h-1}+\nu \hat {g}^{h}$, 0≤*ν*≤1 and $\hat {\beta }^{h}= \hat {\beta }^{h-1}+\nu \hat {\beta }^{s_{h}}$,
5. Repeat steps 2 to 4 until *h*=*h*_*stop*_.

The number of iterations *h*_*stop*_∈[ 0,1000] can be determined by using the AIC criterion.

### Partial least squares (PLS)

PLS is a linear method for dimension reduction in a linear regression setting. It consists in replacing the *p* original variables by *r* orthogonal components, *t*_*h*_, *h*=1,…,*r*, *r*<*p*, so that the covariance between the response and the components is maximum. These components are iteratively calculated as linear combinations of variables *x*_.*j*_, *j*=1,…,*p*

$$t_{h}=\mathbf{X}\omega_{h},\; h=1,\ldots,r $$

where *ω*_*h*_ is a *p*-length vector of weights. These weights are such that they maximize the covariance between the components and the response.

(6) $$\begin{array}{@{}rcl@{}} \omega_{h}=\text{argmax}\; cov^{2}(t_{h},y) \end{array} $$

with *ωh*′*ω*_*h*_=1 and *th*′*t*_*l*_=0, *h*>*l*.

The final model is defined as:

$$\begin{array}{ll} y & =\sum_{h=1}^{r} t_{hi}c_{h}+\epsilon \\ & =\sum_{h=1}^{r} X_{i}\omega_{h}c_{h}+\epsilon \\ & =\mathbf{X}\beta_{PLS}+\epsilon \end{array} $$

where *c*_*h*_ are the regression coefficients in the regression of *y* on *t*_*h*_, *β*_*PLS*_ are the PLS regression coefficients and *ε* the residuals.

Weighted PLS (WPLS) can be used for models suffering from heteroscedasticity. In this case, it is the weighted covariance that is maximized: *cov*^2^(**W**^1/2^*t*_*h*_,**W**^1/2^*y*), with **W** a *n*×*n* weight matrix.

### Ridge partial least squares (RPLS)

The objective of RPLS is to extend the PLS method to a binary response. The main idea is to find a continuous pseudo-response which has a linear relationship with the variables **X** and then perform PLS with this continuous version of *y*. Fort and Lambert-Lacroix [16] suggested replacing *y* with the pseudo-response $\tilde {y}^{*}$ obtained at the convergence of the IRLS algorithm. To ensure the existence of the ML estimates and thus the finite norm of $\tilde {y}^{*}$, they proposed using a ridge version of the IRLS algorithm [23] on the high-dimensional data **X**. It consists in replacing (4) by

(7) $$\begin{array}{@{}rcl@{}} \hat{\beta}_{Ridge}^{h+1}=\left(\mathbf{X}'\mathbf{W}^{h}\mathbf{X}+\lambda \Sigma^{2}\right)^{-1}\mathbf{X}'\mathbf{W}^{h}\tilde{y}^{h} \end{array} $$

where *λ* is the shrinkage parameter and *Σ*^2^ a diagonal matrix [16].

The *β*_*PLS*_ parameters are then estimated using a WPLS on *X* and $\tilde {y}^{*}$ with the weight matrix *W*^∗^ being calculated with the IRLS algorithm.

### RPLS with the clinical predictor as an offset: RPLSOffClin

In this section, we propose to combine clinical and high-dimensional variables using RPLS in a two-step procedure.

The first step consists in fitting a logistic regression model using the clinical variables **Z** only. This leads to the estimation of the logistic regression coefficients $\hat {\gamma }=(\hat {\gamma }_{1},\ldots,\hat {\gamma }_{q})$. The clinical predictor is then defined as $\eta _{\textit {clin}}=\mathbf {Z}\hat {\gamma }$ The objective of the second step is to build the following model

$$y = \eta_{clin} + \mathbf{X}\beta_{\textrm{RPLSOffClin}} $$

where *β*_RPLSOffClin_ are the parameters to be estimated.

To achieve this goal, we propose to introduce the clinical predictor as an offset into the RPLS procedure.

For this, a modified Ridge IRLS algorithm is first used to obtain a pseudo response $\tilde {y}^{*}$ and *W*^∗^ at convergence.

1. Initialization: *h*=0, $\beta _{\textit {Ridge}}^{h}=0$.
2. Until convergence do
  $\eta ^{h}=\eta _{\textit {clin}} + \mathbf {X}\beta _{\textit {ridge}}^{h-1}$
  *π*^*h*^=1/(1+ exp(−*η*^*h*^))
  **W**^*h*^=*diag*[*π*^*h*^ (1−*π*^*h*^)]
  $\tilde {y}^{h}=X\beta _{\textit {ridge}}^{h-1} + \left (y-\pi ^{h}\right)/\mathbf {W}^{h}$
  $\beta _{\textit {Ridge}}^{h}=\left (\mathbf {X}'\mathbf {W}^{h}\mathbf {X}+\lambda \Sigma ^{2}\right)^{-1}\mathbf {X}'\mathbf {W}^{h}\tilde {y}^{h}$
  *h*=*h*+1

Then WPLS was used to estimate the *β*_*RPLSOffClin*_ coefficients with the following objective function:

(8) $$\begin{array}{@{}rcl@{}} \omega_{h}=\text{argmax}\; cov^{2}\!\left(\mathbf{W}^{*1/2}t_{h},\mathbf{W}^{*1/2}(\tilde{y}^{*}-\eta_{clin})\right) \end{array} $$

### Sparse generalized PLS (SGPLS)

Chun and Keles transformed the maximization problem (6) into the following minimization problem:

(9) $$ {}\text{min}_{\omega,c}{-\kappa\omega'M\omega\,+\,(1\,-\,\kappa)(c\,-\,\omega)'M(c\,-\,\omega)\,+\,\delta_{1}|\!|c|\!|_{1}\,+\,\delta_{2}|\!|c|\!|_{2}}  $$

subject to *ω*^′^*ω*=1 and where $M=\mathbf {X}'\mathbf {W}\tilde {y}\tilde {y}'\mathbf {W} \mathbf {X}$ and *κ* is a tuning parameter.

This puts exact zeros in a surrogate weight vector *c* instead of the original weight *ω* by imposing an $\mathcal {L}_{1}$ penalty and by keeping *ω* close to *c*. The $\mathcal {L}_{2}$ penalty controls the multicollinearity problem.

When *Y* is a univariate response, the solution to the problem (9) is given by $\hat {c}=\left (|H|-\delta \text {max}_{1\leq j \leq p}|H_{j}|\right)_{+} sign(H)$ where $H=\mathbf {X}'\mathbf {W} \tilde {y}/||\mathbf {X}'\mathbf {W} \tilde {y}||$ and *δ* is a tuning parameter, with 0<*δ*<1.

In case of a binary response, the SGPLS algorithm is declined as follows:

1. Initialization: *β*=0, $A={\varnothing }$, where *A* denotes the set of active variables.
2. Repeat until convergence: $\Delta \hat {\beta }<\epsilon $
  *η*^*h*^=**X***β*^*h*−1^
  *π*^*h*^=1/(1+ exp(−*η*^*h*^))
  $\tilde {y}^{h}=X\beta ^{h-1}+\left (Y-\pi ^{h}\right)/W^{h}$
  *W*^*h*^=*π*^*h*^(1−*π*^*h*^)
  $\beta ^{h}=\left (\mathbf {X}'\mathbf {W}^{h}\mathbf {X}\right)^{-1}\mathbf {X}'\mathbf {W}^{h}\tilde {y}^{h}$
  Solve the optimization problem (9) and obtain the estimate $\hat {c}$
  Variable selection: $A=\{j: \hat {c}_{j}\neq 0 \}\cup \{j: \hat {\beta }_{j}\neq 0 \}$
  Perform PLS on the selected variables *X*^*A*^
  Update $\hat {\beta }$

### SGPLS with the clinical predictor as an offset: SGPLSOffClin

As for RPLS, we propose a two-step procedure to combine clinical and high-dimensional variables using SGPLS. The first step consists in estimating the clinical parameters *γ* and the second step in estimating the MS parameters *β* using a modified SGPLS algorithm. The clinical parameters are estimated through a logistic regression model. The SGPLS algorithm is modified by replacing step 2a with that of the modified Ridge IRLS algorithm:

2 Until convergence do
  $\eta ^{h}=\eta _{\textit {clin}} + \mathbf {X}\beta _{\textit {ridge}}^{h-1}$
  *π*^*h*^=1/(1+ exp−*η*^*h*^)
  **W**^*h*^=*diag*[ *π*^*h*^ (1−*π*^*h*^)]
  $\tilde {y}^{h}=X\beta _{\textit {ridge}}^{h-1} + \left (y-\pi ^{h}\right)/\mathbf {W}^{h}$

The classic PLS performed in step 2e is replaced by a weighted PLS with the objective function defined in (8).

### Simulated data

A simulation study was conducted to assess the ability of the methods to recover the correct estimates of *γ* and *β* with different numbers of individuals *n* and variables *p* under study.

The following model is considered

$$\eta = \mathbf{Z}\gamma + \mathbf{X}\beta $$

where **Z** (resp. **X**) is the matrix of clinical (resp. high-dimensional) variables and *γ* (resp. *β*) the regression coefficients vector. The number *q* of clinical variables is set to *q*=5, the *γ*-coefficients to *γ*_*j*_=1.5, *j*=1,…,*q* and the number *p*^∗^ of active high-dimensional variables to *p*^∗^=20. The *β*-coefficients are fixed as follows: *β*_*k*_=*μγ*_*j*_, *k*=1,…,*p*^∗^ and *β*_*k*_=0 for *k*=*p*^∗^+1,…,*p*, where *μ* controls the importance of the high-dimensional variables over the clinical ones. The variables *z*_.*j*_, *j*=1,…,*q*, and *x*_.*k*_, *k*=1,…,*p* follow a normal distribution $\mathcal {N}(0,1)$. The response variable *y* follows a binomial distribution of parameters *n* and *π* where *π*=1/(1+*e*^−*η*^).

In our simulations we chose two different cases. Case 1: the clinical variables have more predictive importance than the MS variables, *μ*=0.5. Case 2: the MS variables are more important than the clinical ones, *μ*=2. The sample sizes *n* were chosen as 100, 200, 500. The number of variables *p* was equal to 500. For each (*n*,*p*), 50 datasets were simulated and split into one training dataset (80% of the individuals) and one test (remaining 20% of the individuals) dataset.

### Real data

The real dataset concerns steatosis, which corresponds to an accumulation of fat in the liver. If fatty liver disease is allowed to progress, it will turn into steatohepatitis, a serious inflammation of the liver. If this is not treated, cell damage will begin to occur, potentially putting the patient at risk of death. It is thus of major importance to diagnose steatosis as early as possible. At the moment, steatosis is detected through MRI techniques that may be difficult to bear for some patients. In this study, the goal was to look for proteomics markers that may allow the diagnosis of steatosis through a simple blood test. Samples were collected from the Endocrinology Department of Dijon Teaching Hospital. This single-center study was approved by our regional ethics committee (Protection to Persons and Property Committee, CPP Est II, France). Written informed consent was obtained from all patients prior to inclusion in the study. Between February 2008 and November 2010, consecutive patients were screened prospectively at the endocrinology department for the following inclusion criteria: type 2 diabetes, the absence of acute or chronic disease based on the patient’s medical history, physical examination, and standard laboratory tests (blood counts, electrolyte concentrations); and alcohol consumption of less than 20g/day. Patients who had received thiazolidinediones were excluded. For the purpose of this paper, the groups of patients were defined by the terciles of the distribution of the steatosis rate measured by MRI. Patients belonging to the first third of the set were considered at low risk of steatosis (74 patients), and patients belonging to the last third of the set at high risk of steatosis (76 patients). The related clinical dataset consisted of 7 variables, namely nephropathy, level of gamma-GT, triglycerides, ASAT and ALAT, Body Mass Index and diabetes duration. Datasets were split 100 times into one training dataset (80% of the individuals) and one test (remaining 20% of the individuals) dataset.

Blood samples from each of the patients were analyzed through MALDI-TOF mass spectrometry. After purification using magnetic beads to retain only proteins that had specific biochemical properties, the MS signals were acquired with Xtrem MALDI-TOF *(Bruker Daltonics, Bremen, Germany)* using ground steel target plates with an HCCA matrix. MS signals resulting from MALDI-TOF are contaminated by different sources of technical variations. To eliminate these variations and to extract the biological signal of interest, a prior pre-processing step was applied to the raw data [24-26]: 1) Elimination of the random measurement noise with wavelet methodology. 2) Subtraction of the baseline by adjusting the smoothing cubic spline to local intensity minima. 3) Normalization of spectra using the Total Ion Count (TIC): intensities of each spectrum were divided by the corresponding TIC to allow the comparison of spectra on the same scale. 4) Peak detection which consists in identifying m/z corresponding to potential proteins or peptides of interest. 5) Elimination of the interplate effect by using an empirical Bayes method [27]. All of the pre-processing steps were performed with Rgui software. At the end of these steps, 183 variables were selected as candidate markers.

## Results

### Compared methods

- Clin: logistic regression model using clinical variables only.
- Boost: binomial boosting with component-wise least squares procedure on MS data.
- BoostOffClin: two-step procedure proposed by Boulesteix and Hothorn [18]: 1) estimation of the regression coefficients for clinical variables using a logistic model, 2) estimation of the regression coefficients of the MS variables using a boosting algorithm with the clinical predictor as an offset.
- ClinOffBoost: 1) estimation of a MS predictor using a boosting algorithm. 2) estimation of the regression coefficients of the clinical variables using logistic regression with the MS predictor as an offset.
- SGPLS: SGPLS on MS data.
- SGPLSOffClin: like for method (3), the SGPLS algorithm was run with the clinical predictor as an offset.
- ClinOffSGPLS: the same as method (4), but the MS predictor was estimated using the SGPLS algorithm.
- RPLS: RPLS on MS data.
- RPLSOffClin: like for methods (3) and (6), the RPLS algorithm was run with the clinical predictor as an offset.
- ClinOffRPLS: the same as methods (4) and (7), but the MS predictor was estimated using the RPLS algorithm.

Comparing methods using clinical (Clin) or MS (Boost, SGPLS, RPLS) variables made it possible to evaluate the predictive performances of the two types of variables independently. Comparing methods that combined both clinical and MS data made it possible to determine whether or not combining the two types of data improved the prediction as expected. To determine which of the two types of variables brought additional predictive value to the other, the variables were combined in two different ways. First method: prior estimation of the clinical parameters $\hat {\gamma }$ and then estimation of the MS parameters $\hat {\beta }$ in a model with the clinical predictor as an offset. Second method: prior estimation of the MS parameters $\hat {\beta }$ and then estimation of the clinical parameters $\hat {\gamma }$ in a model with the MS predictor as an offset. Hence the additional predictive value of MS variables was evaluated with BoostOffClin, SGPLSOffClin and RPLSOffClin methods, and the additional predictive value of clinical variables with ClinOffBoost, ClinOffSGPLS and ClinOffRPLS methods.

### Tuning parameters

The glmboost function used to perform the Binomial boosting algorithm is available in the R-package mboost [28]. The step-length factor *ν* was chosen to be small since it has been shown that it improved the predictive accuracy [22]. Bühlmann and Hothorn [12] proposed *ν*=0.1.

The parameter *λ* of the RPLS and RPLSOffClin algorithms was chosen by cross-validation independently for each simulated dataset and for the real dataset with *λ*∈[ 80,200;0.1]. The range for *λ* was chosen empirically.

The 2 parameters *κ* and *δ* of the SGPLS and SGPLSOffClin algorithms were chosen by cross-validation independently for each simulated dataset and for the real dataset, with *r*∈[ 1,5] and *δ*∈[ 0,1;0.1]. The range for *κ* was chosen empirically.

A subject was classified in one group if its probability of belonging to this group was higher than 0.5.

### Performance assessment of the methods

**Simulated data.** In order to evaluate the prediction abilities of the presented models, we calculated the misclassification rate (MCR) as follows: 1- the model was first estimated on the training set, 2- the estimated parameters were then used for prediction on the test set, 3- let $\hat {y}$ be the predicted response and *y* the true response

$$MCR = \frac{card(\hat{y}\neq y)}{card(y)}*100 $$

In case of Boost (2), BoostOffClin (3), SGPLS (5) and SGPLSOffClin (6) the number of true and false positives among selected variables was also estimated. True positives correspond to active variables selected by one model, whereas false positives correspond to non active variables wrongly selected by the model.

**Real data.** Only the MCR was used on the real dataset.

We can note that building significance tests for $\mathcal {L}_{1}$ penalized estimation procedures with *p* larger than *n* is still not clearly understood in the mathematical statistics community (see, for example, the recent work by Lockhart *et al.* [29] and the references therein). Furthermore, the test procedures rely on assumptions on the correlation among the explanatory variables (which must not be too strongly correlated) that are clearly not satisfied in mass spectrometry. For this reason statistical significance tests were not employed in our study.

### Simulated data

In our simulations we chose two different cases, in the first, the clinical variables had more predictive importance than the MS variables and in the second, the MS variables were more important than the clinical ones. Table 1 shows the means and the standard deviations of the MCR over the 50 simulated datasets for case 1 for each of the compared methods. Table 2 shows the same results for case 2. These tables also show the MCR for the Oracle model. The Oracle model was estimated using all of the clinical variables and the active MS variables only.

**Table 1:** **Misclassification rate - case 1**

	***μ*** **=0** ***.*** **5**		
	**n=100**	**n=200**	**n=500**
1) Clin	28 (11)	29 (6)	27 (4)
2) Boost	46 (11)	45 (7)	37 (5)
3) BoostOffClin	30 (11)	27 (7)	18 (3)
4) ClinOffBoost	32 (11)	27 (5)	22 (4)
5) SGPLS	49 (11)	45 (8)	38 (5)
6) SGPLSOffClin	44 (13)	35 (12)	24 (5)
7) ClinOffSGPLS	32 (11)	28 (6)	23 (4)
8) RPLS	47 (12)	43 (7)	40 (4)
9) RPLSOffClin	27 (9)	27 (6)	24 (5)
10) ClinOffRPLS	39 (11)	37 (8)	34 (5)
Oracle	18 (8)	16 (5)	13 (3)

This table contains the mean and the standard deviation of the MCR over 50 simulated datasets for each compared method and for *μ*=0.5 (i.e. lower predictive importance of MS data than clinical data).

**Table 2:** **Misclassification rate - case 2**

	***μ*** **=2**		
	**n=100**	**n=200**	**n=500**
1) Clin	43 (10)	43 (6)	44 (4)
2) Boost	41 (13)	28 (6)	15 (3)
3) BoostOffClin	39 (13)	30 (6)	13 (3)
4) ClinOffBoost	40 (14)	28 (7)	14 (3)
5) SGPLS	41 (11)	32 (8)	14 (3)
6) SGPLSOffClin	41 (10)	36 (8)	14 (4)
7) ClinOffSGPLS	40 (12)	32 (8)	15 (4)
8) RPLS	40 (11)	34 (7)	29 (4)
9) RPLSOffClin	38 (11)	36 (9)	30 (4)
10) ClinOffRPLS	38 (14)	34 (7)	29 (4)
Oracle	15 (8)	8 (4)	6 (2)

This table contains the mean and the standard deviation of the MCR over 50 simulated datasets for each compared method and for *μ*=2 (i.e. higher predictive importance of MS data than clinical data).

### MCR results

A first well-known observation was that the MCR decreases when the number of individuals in the dataset increases and the standard deviations are at least divided by two, meaning that all the procedures performed better for larger sample sizes. Comparing the MCR between cases 1 and 2, we observed that the models involving clinical variables only (resp. MS variables) performed worse (resp. better) when MS variables were more important than when clinical variables were more important. This is consistent with the simulation settings.

Case 1, clinical variables had greater predictive importance than the MS variables. The MCR of Clin (1) was better than the MCR of Boost (2), SGPLS (5) and RPLS (8) which is consistent with the case 1 simulation, in which the clinical variables had greater predictive importance than the MS variables. When the clinical predictor was introduced as an offset in BoostOffClin (3), SGPLSOffClin (6) or RPLSOffClin (9), the MCR was lower than for Boost(2), SGPLS (5) or the RPLS (8). The GLM models, which include the MS predictor as an offset (ClinOffBoost (4), ClinOffSGPLS (7) and ClinOffRPLS (10)), were also more efficient than Boost (2), SGPLS (5) and RPLS (8). As expected, models that combined both types of data were more efficient than those using only MS variables. The information from the clinical variables was correctly used to counterbalance the lack of information obtained from the MS variables. ClinOffBoost (4) and ClinOffRPLS (10) had a lower predictive performance than BoostOffClin (3) and RPLSOffClin (9) suggesting that it is more suitable to use as an offset a predictor that uses only the most informative variables. When *n*=500, BoostOffClin (3), ClinOffBoost (4), SGPLSOffClin (6), ClinOffSGPLS (7) and RPLSOffClin (9) were more efficient than Clin (1) underlining the fact that a large number of individuals is needed to recover the information when the number *p* of variables is high.

Case2, MS variables had greater predictive importance than the clinical variables. When *n* was large enough, all of the models outperformed the Clin (1) model. When Boost (2), BoostOffClin (3), SGPLS (5) and SGPLSOffClin (6) were used to select variables, the MCR was lower than was the case with RPLS (8) and RPLSOffClin (9). The ClinOffBoost (4) and ClinOffSGPLS (7) methods had a better MCR than the ClinOffRPLS (10) method too. These results confirm the advantage of the dimension reduction performed through the variable selection process over the dimension reduction performed by PLS only.

### Variable selection

To evaluate and compare the performance of variable selection performed by Boost and SGPLS, we calculated the true and false positives for each of the methods. The influence of the clinical information on the selection was evaluated by comparing true and false positives obtained for Boost (resp. SGPLS) and BoostOffClin (resp. SGPLSOffClin).

**Figure 1:**
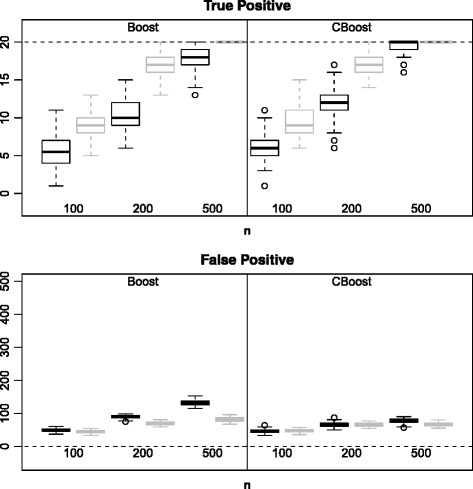
**True and False Positives for the Boost and BoostOffClin methods.** Boxplots representing the distribution of the number of True and False Positives over 50 simulated datasets for the Boost and BoostOffClin methods. The number of variables *p* was equal to 500 and the number of individuals *n* was equal to 100, 200 or 500. Black boxplots correspond to *μ*=0.5 and grey ones to *μ*=2.

Figure 1 presents the true positive (TP) and false positive (FP) results for Boost and BoostOffClin. The number of TP quickly tended towards the true number of active variables (*p*^∗^=20) as *n* increased, and was higher in case 2 than case 1. The number of FP was lower for BoostOffClin than for Boost in both cases. Hence, the high predictive importance of the clinical variables allows better selection of variables. When *n* was large enough, the BoostOffClin algorithm was able to select all the active variables.

**Figure 2:**
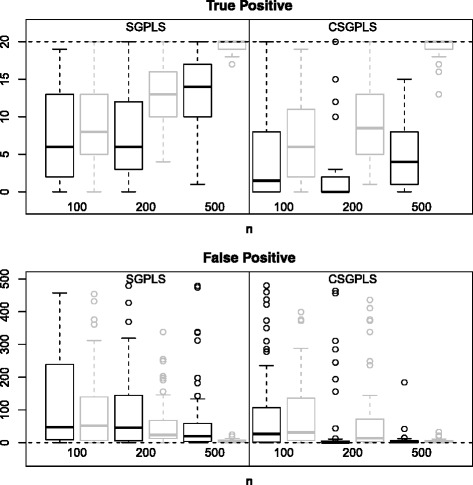
**True and False Positives for the SGPLS and SGPLSOffClin methods.** Boxplots representing the distribution of the number of True and False Positives over 50 simulated datasets for the SGPLS and SGPLSOffClin methods. The number of variables *p* was equal to 500 and the number of individuals *n* was equal to 100, 200 or 500. Black boxplots correspond to *μ*=0.5 and grey ones to *μ*=2.

Figure 2 represents TP and FP results for SGPLS and SGPLSOffClin. The dispersion of the number of TP was very wide and the number of TP was much lower than 20 except in case 2 when *n*=500. It was more difficult for SGPLSOffClin than for SGPLS to identify the active variables. The number of FP decreased and tended to 0 as *n* increased.

SGPLS and SGPLSOffClin selected few non-active variables but found it difficult to identify active variables. Although the number of FP was slightly higher with the Boost methods than with the SGPLS methods, the number of TP was much better. In parallel Tables 1 and 2 show that Boost and BoostOffClin MCR were lower than SGPLS and SGPLSOffClin MCR.

### Real data

**Figure 3:**
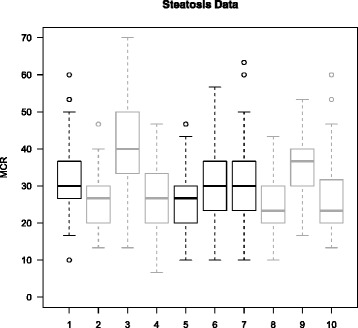
**Misclassified rate for the steatosis dataset.** Boxplots representing the missclassification rate distribution over 100 random test datasets for the steatosis dataset. Methods: 1. Clin, 2. Boost, 3. BoostOffClin, 4. ClinOffBoost, 5. SGPLS, 6. SGPLSOffClin, 7. ClinOffSGPLS, 8. RPLS, 9. RPSLSOffClin, 10. ClinOffRPLS.

Figure 3 presents the distribution of the MCR for the different methods in the case of the Steatosis dataset. The Clin model (1), which included only clinical variables, was less efficient than models with only MS variables (Boost (2), SGPLS (5) and RPLS (8)). This seems to indicate that MS variables have more predictive information than clinical variables. Most of the methods that combine clinical and MS variables perform as well as methods that use only one kind of information. The MCR for the BoostOffClin (3) and RPLSOffClin (9) methods were slightly higher than the others, which was an unexpected findings, as the results on simulated datasets showed an improvement when both clinical and MS variables were used in the model. By taking into account standard deviations, these results were at least as good as those provided by the Clin model (1). It is hard to decide which method is the best one. This can be explained by the size of the Steatosis dataset. Indeed, we observed on simulated datasets that the methods provided similar results when the sample size was below 200. However, all of the methods performed quite well with a mean MCR equal to 30%, suggesting the presence of potential biomarkers.

## Discussion

To better understand the previous results, we were interested in the predictive part of the predictor *η* not explained by the clinical variables. This quantity was obtained by projecting *η* onto the orthogonal of the subspace spanned by the clinical variables *PZ*$\bar {c}=\frac {\Vert (I-PZ)\eta \Vert }{\Vert \eta \Vert }$

**Figure 4:**
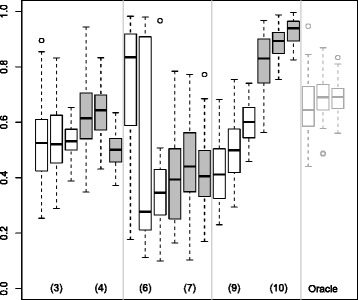
**Predictive part of the predictor** ***η*** **not explained by the clinical variables for case 1 - simulated data.** Boxplots representing the distribution of the part of the predictor not explained by the clinical variables $\bar {c}$ for 50 simulated test sets in case 1 (when the clinical variables carry more information than the MS variables). Methods: 3. BoostOffClin, 4. ClinOffBoost, 6. SGPLSOffClin,7. ClinOffSGPLS, 9. RPSLSOffClin, 10. ClinOffRPLS.

**Figure 5:**
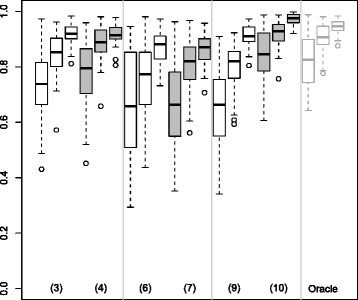
**Predictive part of the predictor** ***η*** **not explained by the clinical variables for case 2 - simulated data.** Boxplots representing the distribution of the part of the predictor not explained by the clinical variables $\bar {c}$ for 50 simulated test sets in case 2 (when the clinical variables carry less information than the MS variables). Methods: 3. BoostOffClin, 4. ClinOffBoost, 6. SGPLSOffClin, 7. ClinOffSGPLS, 9. RPSLSOffClin, 10. ClinOffRPLS.

**Figure 6:**
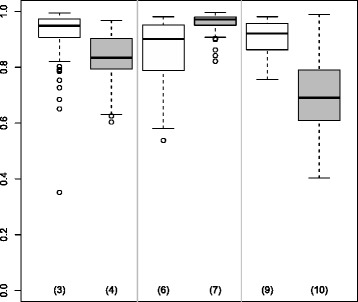
**Predictive part of the predictor** ***η*** **not explained by the clinical variables for the steatosis dataset.** Boxplots representing the distribution of the part of the predictor not explained by the clinical variables $\bar {c}$ for 100 test sets for the steatosis datasets. Methods: 3. BoostOffClin, 4. ClinOffBoost, 6. SGPLSOffClin, 7. ClinOffSGPLS, 9. RPSLSOffClin, 10. ClinOffRPLS.

Figures 4 and 5 show the distribution of the $\bar {c}$ values computed for the 50 test sets and for the simulated datasets in case 1 and 2, respectively. Figure 6 shows the distribution of the $\bar {c}$ values for the real dataset.

In Figure 4 (when the clinical variables carry more information than the MS variables), the $\bar {c}$ distribution for the BoostOffClin (3) and ClinOffBoost (4) methods were close. The $\bar {c}$ for the SGPLSOffClin method (6) was higher than that for ClinOffSGPLS (7). The $\bar {c}$ value for the RPLSOffClin method (9) was lower than that for the ClinOffRPLS method (10). When comparing results for $\bar {c}$ and MCR, we can see that their distribution evolved in the same way. The greater the amount of clinical information in the predictor, the more efficient the prediction. In Figure 5, the information was carried by the MS variables. In this case, the $\bar {c}$ value was high for all of the methods and lower than in case 1, which is consistent with our simulation settings. In both cases Boost methods gave values that were the closest to the Oracle model.

Figure 3, which presents the MCR results for the real dataset, shows that models that included only MS variables (2, 5, 8) were more efficient than the Clin model (1). This suggests that MS variables contain more predictive information than clinical variables. The $\bar {c}$ distribution observed in Figure 6 for the real dataset was similar to that in Figure 5 for the simulated data in case 2 with *n*=100. This confirms that there was more information in the MS variables than in the clinical variable.

Among the variety of ways to evaluate the predictive accuracy of the considered methods we chose to use the MCR. This criterion is simple to compute and interpret and is widely used for this purpose. Other criteria could have been considered, such as ROC curves, which are also suitable for evaluating and comparing the performances of classification models when the response variable is binary. Even if we believe that it would not have changed the main conclusions, the choice of the MCR rather than the ROC curve could be a limitation of our study.

## Conclusion

The identification of new diagnostic or prognostic biomarkers is one of the main aims of clinical research. The large number of variables compared to the number of individuals included in the study is a statistical challenge. In this work, we have evaluated and we have compared the predictive power of new and existing methods for binary classification in models using clinical data, MS data or a combination of both. The proposed methods meet the challenge of high-dimensional data analysis, including the question of the selection of predictive MS variables. This was performed using various dimension reduction methods and penalization methods. The evaluation of the methods on simulated datasets revealed the first quite logical observation: all of the methods performed better for larger sample sizes. When the sample size was small, the misclassification rate and its dispersion were too high to ensure fair comparison of the methods. The dispersion and the mean of the misclassification rates were much lower when *n* was large. This recalls the importance of working with samples of sufficient size. Mainly, it highlights that even with methods dedicated to high-dimensional data analysis, it is a challenge to recover the true information contained in the datasets. The second observation was that the combination of both clinical and MS variables made it possible to outperform methods that used only one of the two kinds of variables. The methods that included variable selection like boosting and SGPLS were more efficient than methods without variable selection (RPLS). Variable selection using Boost was better than that using SGPLS. Boosting not only selected the true active variables, but the misclassification rate was also lower. Despite good predictive power on the real dataset, it is hard to compare the predictive efficiency of the methods. According to the results of the simulation study, this was the case when working with small sample sizes. However, it is worth noting that with a sample size of 150 individuals, the real dataset we used was a classical example of datasets available in clinical research when it comes to high-dimensional analysis. In fact, the identification of new diagnostic or prognostic markers is still a challenge, due to this high-dimensional setting. Few studies have given robust and validated results because of the difficulty to conduct large enough studies. We may consider the interest of making technologies evolve to study ever more variables at the same time if the statistical methodologies are not able to give robust results with too few individuals under study, which is often the case because of budget constraints.

Keeping this in mind, to ensure good prediction, we recommend working with a large enough dataset, performing variable selection, and whenever possible, combining clinical and MS variables.
